# Mitochondria, Oxidative Stress and Innate Immunity

**DOI:** 10.3389/fphys.2018.01487

**Published:** 2018-10-18

**Authors:** Yuxin Chen, Zhongyang Zhou, Wang Min

**Affiliations:** ^1^Department of Laboratory Medicine, Nanjing Drum Tower Hospital, Nanjing University Medical School, Nanjing, China; ^2^Center for Translational Medicine, The First Affiliated Hospital, Sun Yat-sen University, Guangzhou, China; ^3^Department of Pathology and the Vascular Biology and Therapeutics Program, Yale School of Medicine, New Haven, CT, United States

**Keywords:** mitochondria, innate and adaptive immune response, inflammasome, ROS, thioredoxin

## Abstract

Canonical functions of mitochondria include the regulation of cellular survival, orchestration of anabolic and metabolic pathways, as well as reactive oxygen species (ROS) signaling. Recent discoveries, nevertheless, have demonstrated that mitochondria are also critical elements to stimulate innate immune signaling cascade that is able to intensify the inflammation upon cytotoxic stimuli beyond microbial infection. Here we review the expanding research field of mitochondria and oxidative stress in innate immune system to highlight the new mechanistic insights and discuss the pathological relevance of mitochondrial dysregulation induced aberrant innate immune responses in a growing list of sterile inflammatory diseases.

## Introduction

Mitochondria are multifunctional organelles that collaborate with their host cells in biosynthesis, metabolism, and cell death or survival functions. Recent growing evidence has highlighted expanded roles of mitochondria in modulating oxidative stress as well as innate immune responses. Since mitochondria is the major resource of reactive oxygen species (ROS) that emerges as a convergent signaling hub that regulates diverse developmental, environmental, and pathological stimuli. Along with mitochondrial dysfunction and excessive ROS observed in various disease conditions including cardiovascular diseases, autoimmune diseases, metabolic syndromes as well as tumor, aberrant innate immune responses were also identified, which is considered to be a common factor which drives the inflammatory pathology of these conditions. In this review, we present a comprehensive landscape regarding the role of mitochondria in innate immunity. We also detail recent advances in understanding novel function of mitochondria in inflammasome machinery, highlighting their emerging roles in driving the sterile inflammatory responses upon injured or damaged tissues and cells.

## Mitochondrial Function and ROS

Mitochondria are ubiquitous multifunctional organelles with a double-membrane structure, which are present in majority of mammalian cell types. They are dynamic, branched networks with continuous cycles of fission and fusion (Mishra and Chan, 2014). Mitochondria bear the residual genome (mitochondrial DNA, mtDNA) that is essential for their activity of oxidative phosphorylation (OXPHOS) and a protein library containing with 1,200 proteins (Calvo and Mootha, 2010) that are varied substantially between cell and tissue types (Scarpulla, 2008). It is believed that mitochondrial biogenesis and homeostasis, including mtDNAs, are under tight regulation of nucleus. This needs bi-directional signaling pathways that mediate crosstalk between the nucleus and mitochondria (Shadel and Horvath, 2015). Such mitochondrial retrograde signaling pathways are highly conserved from prokaryotic to eukaryotic cells which potentially can trigger both favorable and maladaptive responses. Therefore, the number, morphology, distribution, and activity of mitochondria are constantly altered in respond to physiological, developmental, and environmental stimuli.

As the sites of the tricarboxylic acid (TCA) cycle and OXPHOS, mitochondria produce substantial ATP using the electrochemical gradient by the electron transport chain (ETC), whereas mitochondria could also produce ROS predominately at the ETC complex I and complex III (Murphy, 2009). ROS were initially considered to exert damage-promoting, detrimental effects; however, recent studies have depicted it as an emerging central signaling molecule. Therefore, beyond the traditional roles of mitochondria in metabolism such as glucose oxidation as well as biosynthesis of fatty acid, amino acid and hormones, mitochondria are also actively involved in ROS signaling, apoptosis and innate immunity (Shadel and Horvath, 2015).

Given its intrinsic complicated nature of mitochondria, mitochondrial dysfunction can induce distinct stress signals. For example, reduced OXPHOS and ETC activity can result in disturbed mitochondrial ROS (mtROS) production, eliminated mitochondrial membrane potential, or reduced cellular adenosine 5′-triphosphate (ATP) or energy (Butow and Avadhani, 2004; Sena and Chandel, 2012). As the major generator of ROS, mitochondria are also prone to become the target of ROS followed by pathological consequences. For example, elevated free radicals associated with mtDNA oxidative damage trigger cell apoptosis by inducing mitochondrial stress and downstream signaling (Scheibye-Knudsen et al., 2015; West et al., 2015). Finally, dynamic morphology and distribution of mitochondria within cells can also elicit distinct forms of stress that are associated with mitochondrial elimination by mitophagy or autophagy (Labbe et al., 2014). However, mitochondria contain potent anti-oxidant systems that protect mitochondria from ROS-mediated damages, including mitochondrial superoxide dismutase (SOD2) and the thioredoxin system made of thioredoxin-2 (Trx2), thioredoxin reductase-2 and peroxidase 3.

One of the most studied mitochondrial protein is Trx2, a small redox protein with double redox-active sites (C90 and C93). Trx2 is ubiquitously presented in tissues with high metabolic activity, including liver, brain and heart. Trx2 is able to maintain cell in a reduced state by reversible oxidation to Cys disulfide (Trx-S2) via the transfer of reducing equivalents from the catalytic site Cys residue to a disulfide protein substrate (protein-S2). By such way, Trx2 regulates a large number of apoptosis related molecules and critical transcription factors, such as apoptosis signal-regulating kinase 1 (ASK1) (Zhang et al., 2004) and nuclear factor kappa B (NF-κB) (Hansen et al., 2006). Trx2 depletion leads to cytosolic release of cytochrome c from mitochondria and subsequently activation of caspase-3 and -9. Moreover, Trx2 is able to attenuate tumor necrosis factor (TNF)-dependent elevated mtROS and apoptosis (Zhang et al., 2004; Hansen et al., 2006), suggesting that Trx2 modulates TNF-dependent redox signaling in mitochondria (Benhar et al., 2008). Trx2 not only protects against oxidative stress in mitochondria, but also induces the cells insensitive in response to ROS-induced apoptosis. In femoral artery ligation model, mitochondrial Trx2 in the endothelium cells is able to inhibit ASK1-induced apoptosis by elimination of ROS to increase nitric oxide (NO) bioavailability and inhibition of ASK1 activity (Zhang et al., 2004, 2007; Dai et al., 2009). Moreover, a similar role of Trx2 in cardiomyocytes was also identified. The absence of Trx2 in cardiomyocytes exhibits the disorganized mitochondrial arrays and swelling as well as impaired ATP generation, whereas ASK1 is required for Trx2 deletion triggered apoptosis, dysfunctional mitochondria, excessive ROS production observed in cardiomyocytes. Our findings in small animal model were further also validated in the clinical samples (Huang et al., 2015). Reduced Trx2 expression, elevated levels of phosphorylated ASK1 and activated caspase-3 were found in cardiomyocytes of patients with dilated cardiomyopathy (DCM) compared to that of healthy organ donors, indicating human Trx2 is also required to inhibit ASK1-dependent apoptosis signaling. Taken together, as exemplified by Trx2, mitochondrial proteins are capable to modulate cellular activities by maintaining cellular redox state and limiting ROS production.

Recent evidence has highlighted the essential role of mitochondria in activities of immune cells. Mitochondrial physiology, morphology, and metabolism tightly regulate immune cell fate during immune responses. For example, T cell development toward memory or effector phenotypes is tightly modulated by fission and fusion activities of mitochondria (Buck et al., 2016). OXPHOS and ROS production is required for T cell activation, while activated T cells can use either OXPHOS or glycolysis for proliferation. T cell mitochondrial dysfunction has been considered as a signature for infectious diseases and some autoimmune disease. During hepatitis B virus (HBV) infection, various cellular processes centered on mitochondrial activities and ROS were substantially downregulated in CD8 T cells, which might contribute to functional exhaustion of HBV CD8 T cells. Mitochondrion targeted antioxidants can elicit a notable improvement and restore anti-HBV CD8 function (Fisicaro et al., 2017). Mitochondrial hyperpolarization, ATP depletion and elevated ROS production has been observed from T cells from systemic lupus erythematosus (SLE) patients (Gergely et al., 2002), which might result from abnormal NO generation derived from monocytes (Nagy et al., 2003). CD4+ T cells from rheumatoid arthritis patients also exhibited elevated autophagy, ATP depletion, and impaired redox status (Yang et al., 2013, 2016). CD4+ T cells from multiple sclerosis patients displayed remarkable abundance in mitochondrial inner membrane lipid cardiolipin (Vergara et al., 2015). Potential therapeutic strategies include the inhibition of the mitochondrial oxidation and glycolytic rate to restore CD4+ T cell function in autoimmune diseases (Yin et al., 2015).

Beyond immune cells, mitochondrial dysfunction also extensively influences the function of non-immune cells. Taking endothelial cells for example, a representative mechanism that links to sterile inflammation is the so-called senescence associated secretory phenotype (SASP) (Tchkonia et al., 2013). It has been shown that upon mitochondrial dysfunction, endothelial cells secrete multiple pro-inflammatory cytokines includes interleukin (IL)-1, IL-6, and TNF-α, and upregulate intercellular adhesion molecule-1 (ICAM-1) expression which attracts monocyte activation and adhesion (Choi et al., 2018). Indeed, our recent unpublished data have also revealed that Trx2-deletion induced mitochondrial dysfunction also lead to endothelial cell senescence and SASP *in vivo* and *in vitro*, which might further recruit innate immune cells to augment inflammatory responses.

## The Activation of Innate Immune Responses by Mitochondria-Derived ROS

Innate immunity provides a front line of host defense through direct engagement of pathogen or environmental insult, which further initiates the development of an adaptive immune response. However, the ability of innate immune system to resist a subsequent challenge with either the same or a different insult remains unaltered. Although the innate immune system lacks the fine specificity of adaptive immunity that is necessary to produce immunological memory, it can distinguish self from non-self by pattern recognition receptors (PRRs), germline-encoded receptors located at the cell surface and within endosomes (Kawai and Akira, 2006), serving as a sensor to monitor signs of infection or tissue injury. Such evolutionarily ancient PRRs sense a wide range of exogenous pathogen-associated molecular patterns (PAMPs) and endogenous danger-associated molecular patterns (DAMPs). DAMPs include ROS, heat shock proteins, oxidized lipoproteins, and cholesterol crystals. DAMPs could trigger sterile inflammation by binding with PPRs such as Toll-like receptors (TLRs), nucleotide-binding and oligomerization domain (NOD)-like receptors (NLRs), retinoic acid–inducible gene (RIG)-I-like receptors (RLRs), and absent in Melanoma (AIM) 2-like receptors (ALRs) (Sellge and Kufer, 2015). Engagement of these receptors further recruit immune cells to the site of infection or injury in an attempt to clear pathogens or amply inflammatory responses within the host.

**FIGURE 1:**
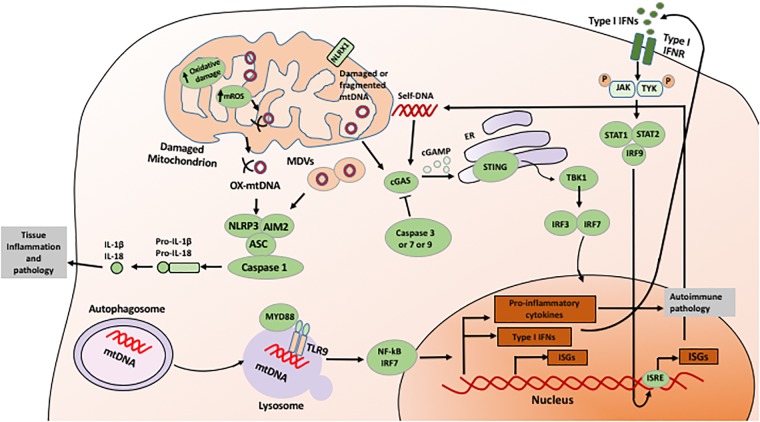
Mechanism of mitochondria-mediated signaling in innate immunity. Mitochondria-derived ROS and released mtDNA directly induces activation of innate immune responses, including activation of inflammasome, sGAS-STING, and NF-kB signaling pathways. See text for details.

The innate immune receptors might also modulate OXPHOS, mitochondrial function (**Figure 1**), ROS and apoptosis present in tissue injury. For example, NLR family member X1 (NLRX1) is a designated innate immune receptor localized in mitochondria. During ischemia-reperfusion injury, NLRX1 is capable to protect against mortality by attenuating mitochondrial impairment and oxidative stress-induced apoptosis of epithelial cells (Stokman et al., 2017). By this way, the innate immune receptor could also control the metabolism of epithelial cells under cytotoxic stress.

An essential member of downstream cytosolic surveillance is the inflammasome, a large multimolecular complex that intensifies the inflammation when sensing microbial components and endogenous danger signals (Kanneganti et al., 2007; Takeuchi and Akira, 2010). The activated inflammasome modulates the proteolytic enzyme caspase-1, result in the maturation of proinflammatory cytokines, IL-1ß and IL-18 (Martinon et al., 2002; Kayagaki et al., 2011). To date, at least two major types of inflammasomes were identified, NLR families and the ALR families (Lamkanfi et al., 2007). NLRs, including the NLRP1, NLRP3 and NLR family caspase recruitment domain (CARD) domain containing 4 (NLRC4) inflammasomes, are characterized by a central located NOD domain that is flanked at N-terminal CARD, pyrin domain (PYD), acidic transactivating domain, or baculovirus inhibitor repeat (BIR) (Franchi et al., 2009; Sutterwala et al., 2014; Tschope et al., 2017). These motifs are capable to bound NLRs with adapter proteins and effectors such as apoptosis-associated speck-like protein containing a CARD (ASC) and caspase-1. C-terminal of NLRs contain leucine-rich repeat (LRR) motifs that regulate NLR activity (Lamkanfi et al., 2007). AIM2 inflammasome, including AIM2 and interferon-gamma inducible factor 16 (IFI16), contains a prototypical DNA binding HIN200 domain and an amino-terminal pyrin motif, through which AIM2 recruits ASC and caspase-1.

The assembly and activation of these inflammasomes are dependent on specific pathogens and endogenous insults (Kanneganti, 2010). For example, the NLRC4 inflammasome is activated in response to bacterial infection such as *Salmonella typhimurium* (Zhao et al., 2011). The NLRP1b and NLRP12 inflammasomes are triggered by anthrax lethal toxin and *Y. pestis* infection, respectively (Levinsohn et al., 2012; Vladimer et al., 2012). NLRP6 is widely present in the intestine that is critical to maintain the intestinal homeostasis (Elinav et al., 2011), whereas NLPR7 inflammasome has been shown to recognize diacylated lipopeptides in human macrophages (Khare et al., 2012). Compared to the NLR family members mentioned above, NLRP3 inflammasome is the most well characterized inflammasome. NLRP3 inflammasome is activated by diverse stimuli including both RNA and DNA viruses, presence of cytosolic bacterial RNA during infection, injury-induced stress molecules such as ROS, ATP and the release of mitochondrial DNA, and harmful environmental substances such as silica and asbestos, K^+^ efflux, and lysosomal destabilization (Piccini et al., 2008; Martinon et al., 2009; Broz et al., 2010; Franchi et al., 2012). Activation of NLRP3 requires two-step signals: firstly priming with either TLR or NLR ligands to enhance NF-κB-driven transcriptional level of NLRP3, and subsequently exposing to microbial toxins and ionophores or endogenous alarmins to trigger inflammasome assembly (Tschopp and Schroder, 2010). Meanwhile, during microbial infection of macrophages, two-step signaling required by NLRP3 inflammasome activation may occur simultaneously (Tschopp and Schroder, 2010). Interestingly, although adenoviral DNA triggers assembly of the NLRP3 inflammasome activation, upon exposure with transfected non-viral cytosolic DNA, caspase-1 activation and IL-1β secretion, however, was dependent on ASC rather than NLRP3 (Muruve et al., 2008). Given such diverse stimuli NLRP3 recognizes, it is believed that the NLRP3 inflammasome was able to recognize cytosolic nucleic acids and other endogenous danger signals indirectly. Indeed, it has been suggested that NLRP3 activation might be triggered by perturbed cell membrane (Barlan et al., 2011).

Given diverse stimuli converge on the activation of NLRP3 including ROS, here we hypothesize that ROS might be the direct mediator to trigger NLRP3. Crystal structural analysis of NLRP3 has identified an unexpected, however, highly conserved disulfide bond between the PYD motif and the nucleotide-binding site motif, that is super sensitive to disturbed redox status (Bae and Park, 2011), indicating a crucial redox role for NLRP3. Indeed, ROS is identified as a potent endogenous ligand for NLRP3, since the early reports has demonstrated that the inhibition of nicotinamide adenine dinucleotide phosphate (NADPH) oxidase-induced ROS inhibited NLRP3 activation in macrophages treated with ATP (Cruz et al., 2007); meanwhile, the absence of the p22*phox* subunit within NADPH oxidase substantially attenuated IL-1β production when macrophages exposed with asbestos (Dostert et al., 2008). In addition to NADPH oxidase-derived ROS, mtROS has also demonstrated to elevate NLRP3 activation (Bulua et al., 2011; Zhou et al., 2011; Wu et al., 2013). Interestingly, NLRP3 is present in the cytosol and endoplasmic reticulum (ER) during rest state but is migrated to mitochondria upon activation, which is mediated by the association of mitochondrial anti-viral signaling protein (MAVS) (Subramanian et al., 2013). Specific inhibition of mtROS, instead of the inhibition of NADPH oxidase, was able to prevent inflammasome-dependent IL-1β expression triggered by cyclic stretch in alveolar macrophage (Wu et al., 2013).

## The Activation of Innate Immune Responses by Mitochondria-Derived DNA

The innate immune system is ultrasensitive to dsDNA. Cellular sources of immune stimulatory self-DNA include nucleus DNA, mtDNA, and DNA from phagolysosomal compartments. Mitochondria in most cells have approximately 2–10 copies of mtDNA, characterized as circular, closed, dsDNA with size of 15,000 base pairs (Kukat and Larsson, 2013). Upon mitochondrial stress induced apoptosis, Bcl2 antagonist/killer (BAK) and Bcl2-associated X protein (BAX) induce permeabilization of mitochondrial outer-membrane (MOMP), leading to cytochrome c release and activation of apoptotic caspases. During this process, mitochondrial network is compromised and newly appeared BAK/BAX macropores allows mitochondrial matrix component containing mtDNA leakage into the cytosol. Similar to NLR inflammasomes, ALR inflammasomes also induce caspase-1 activation and IL-1β cytokine maturation. However, AIM2 inflammasome has been identified as a cytosolic receptor for DNA, including intracellular bacteria *F. tularensis* and mouse cytomegalovirus (Fernandes-Alnemri et al., 2010); while AIM-2 related inflammasome, IFI16 is in the nucleus, which has also implicated in forming an inflammasome complex during herpes simplex virus infection of endothelial cells (Kerur et al., 2011). Contrast with NLR inflammasomes, ALR inflammasomes is able to bind their ligand directly, dsDNA. Further, AIM2 and IFI16 lack CARD domains and recruitment of ASC is required for their activation. Of note, AIM2 is the first inflammasome where a direct receptor:ligand interaction has been formally demonstrated (Fernandes-Alnemri et al., 2009; Hornung et al., 2009). Intriguingly, AIM2 recognizes cytosolic DNA with a minimum length of 80 base pairs in a sequence-dependent fashion (Rathinam et al., 2012). When DNA binding with its C-terminal HIN200 domain, AIM2 undergoes oligomerization and further engages caspase-1 via ASC and secretes IL-1β. In Aim2-deficient mice, it has been shown that the AIM2 inflammasome plays a non-redundant role to induce defense responses against DNA viruses and intracellular bacterial infections (Fernandes-Alnemri et al., 2010). Moreover, AIM2 contributes to the adjuvanticity of DNA vaccines (Suschak et al., 2015) and enhance autoimmune disorders such as SLE via host DNA recognition (Panchanathan et al., 2011). During Nelfinavir treatment, an HIV aspartyl protease, AIM2 could also be activated upon DNA release from the nucleus with a compromised nuclear envelope integrity (Di Micco et al., 2016). Until recently, there is a few reports suggesting a role of AIM2 in caspase-1 activation trigger by mtDNA. The depletion of AIM2 in bone marrow-derived macrophages (BMDMs) leads to reduced IL-1β with mtDNA transfection compared to control BMDMs (Nakahira et al., 2011). A recent study elegantly demonstrated that cholesterol overload causes impaired mitochondrial metabolism and mtDNA release, which further triggers AIM2 inflammasome in activated BMDMs (Dang et al., 2017).

NLRP3 inflammasome could also been directly activated by mtDNA. This was first identified by the observation that the autophagic proteins were able to regulate NLRP3-dependent inflammation by maintaining mitochondrial integrity (Nakahira et al., 2011). Upon stimulation of lipopolysaccharide (LPS) and ATP, the deficiency of autophagy proteins beclin 1 and LC3B in BMDMs leads to dysfunctional mitochondrial and cytosolic translocation of mtDNA. The later event was strictly relied on NALP3 inflammasome activation and mtROS, ultimately enhancing IL-1β and IL-18 secretion. A subsequent study by Shimada et al. (2012) suggested that the essential role of mROS during the process of NLRP3-dependent cytosolic release of mtDNA might be contributed by the intrinsic characteristics of NLRP3, which preferentially binds oxidized mtDNA and further stabilizes it in the cytoplasm after release.

Cyclic GMP-AMP synthase (cGAS) is a prominent cytosolic DNA sensor (Gao et al., 2013). The nucleotidyl transferase enzyme cGAS detects cytoplasmic DNA and produces the second-messenger, cyclic AMP-GMP (cGAMP), which is associated with and subsequently activates stimulator of interferon gene (STING) (Chen et al., 2016). Activated STING is able to recruit TANK-binding kinase 1 (TBK1), which phosphorylates interferon regulatory factor 3 (IRF3) to enhance its homodimerization and migrate into the nucleus, followed by induction of interferon β (IFNβ) and interferon-stimulated genes (ISGs) (Schoggins et al., 2014; Storek et al., 2015; Watson et al., 2015). Despite cGAS-STING signaling pathway has considered as a major defense mechanism against microbial infection, it is also essential to orchestrate type I interferon (IFN) and proinflammatory responses to self-DNA, driving type I IFN induction and ultimately resulting in autoimmune responses as well as antitumor immunity (Barber, 2015; Crow and Manel, 2015; Gray et al., 2015; Roers et al., 2016). Genetic studies in humans have demonstrated that mutations in DNA nucleases such as three prime repair exonuclease 1 (Trex1) lead to cytosolic DNA accumulation which activates the cGAS–STING pathway (Rice et al., 2015). With exposure of genotoxic stress, the collapsed micronuclear envelope help cGAS get access to the damaged nuclear DNA followed by activation of cGAS–STING pathway (Harding et al., 2017; Mackenzie et al., 2017). Interesting enough, it has been demonstrated that mtDNA leaked into the cytosol can activate the cGAS–STING pathway and type I IFN production in the absence of active caspases (West et al., 2015; McArthur et al., 2018).

Here comes an interesting question: since there are billions of cells that undergo apoptosis per day in our body, during which DNA is released into cytosol, how the immune system effectively keep immunologically silent from apoptosis triggered stimuli? A recent unexpected finding is that mitochondria and downstream caspases can determine the immunological status of cell death (Rongvaux et al., 2014). It has long been considered that the highly regulated caspase-dependent apoptosis is immunologically silent, in which the effector caspases including caspase-3, -7, and -9, were necessary to inhibit mtDNA triggered activation of STING (White et al., 2014; West et al., 2015). In contrast, cell death independent of caspase provokes an inflammatory response through releasing DAMPs into the local microenvironment. The activation of such DAMPs further recruits inflammatory cells such as granulocytes, monocytes, and macrophages. Specifically, without the presence of active caspases, Bax and Bak induced MOMP leads the activation of mitochondrial DNA-dependent cGAS–STING pathway followed by the potent induction of type I IFNs and a state of viral resistance. These unexpected mechanistic findings indicated an essential role of mitochondria and caspases, not only on the decision of the cell fate but also on the choice decease in an inflammatory or immunologically silent manner.

It worth mentioning that mtDNA uniquely affects the innate immunity distinct from NADPH oxidase-induced ROS and mtROS. NADPH oxidase-induced ROS serves as an alarm signal in the cytosol that induces efficient defense signal transduction pathways that use hydrogen peroxide as secondary messenger. Although NADPH oxidase-induced ROS could be partially scavenged by a versatile antioxidant system, it also results extensive cellular damage and necrosis. NADPH oxidase-induced ROS can exert oxidative damage on cellular proteins, lipids and nucleic acids, nevertheless, they are also crucial secondary messengers in innate immune responses. ROS is required for heightened sensing by innate immune receptors including TLRs, RLRs, and NLRP3 in an indirect manner via interaction with and modification of other molecules such as mtDNA (Banoth and Cassel, 2018). As reviewed above, mtROS directly contribute to inflammatory cytokine production and innate immune responses. mtROS can directly activate adenosine monophosphate-activated protein kinase (AMPK) and mitogen-activated protein kinase (MAPKs) (Bulua et al., 2011). Phosphorylation of their substrate can directly affect diverse metabolic pathways, regulation of gene expression by transcription factors, and direct activation or inhibition of specific target proteins including innate immune molecules such as RLRs (Tal et al., 2009) and NLRP3 (Zhou et al., 2011). In the absence of any mtROS, mtDNA upon releasing into cytoplasm, extracellular space or circulation during cell death or mitochondrial damage could be sensed by multiple PRRs including cGAS-STING, TLR9, NLRP3, NLRC4, and AIM2 in cell-type and context-dependent manners to trigger pro-inflammatory and type I IFN responses.

## Mitochondria-Mediated Innate Immune Responses in the Context of Sterile Inflammation Related Diseases

Recent studies have illustrated a deleterious role of innate immunity in sterile inflammation, which we have summarized in **Table 1**. Tissue injury would result in cell death accompanied with nucleus and mitochondrial DNA damages. Recently, AIM2 has been shown to be involved in ionizing radiation induced hematopoietic syndrome and severe injury to gastrointestinal (GI) tract. During this process, AIM2 is capable to recognize nucleus DNA damage triggered by radiation and caspase-1 dependent death of bone marrow cells and intestinal epithelial cells (Hu et al., 2016). Blockage of AIM2 inflammasome activity might be an effective therapeutic regimen especially for cancer patients suffering from hematopoietic or GI toxicity. Similarly, using permanent coronary ligation after myocardial infarction (MI) in mice, King et al. (2017) reported a harmful role of IRF3-dependent innate immune response on ventricular remodeling, which supports the key molecules in innate immunity as a new target for protection against MI. Mechanistically, during MI a great number of dying ischemic cell from the heart catastrophically releases large quantities of DAMPs, such as self-DNA, that trigger the innate immune response. Phagocytic macrophages in the heart trigger a fatal response against MI by sensing DNA via cGAS, followed by IRF3-IFN signaling axis activation. Secreted type I IFNs is able to further amplify the response through ISGs by diffusing to the local microenvironment and signaling to cells with interferon-α/β receptor (IFNAR). Indeed, in contrast to wild type (WT) mice after MI, mice with genetic deficiency of cGAS, IRF3, or IFNAR suffer less ventricular dilation and rupture, greater contractile ability and improved survival time. Further, in cardiomyocytes, mitochondrial DNA that escapes degradation specifically activate TLR9-mediated inflammatory response, resulting in exacerbated heart failure (Oka et al., 2012). These studies elegantly demonstrate the molecular inflammation events during pathogenesis of myocardial disease.

**Table 1:** Summary of sterile inflammation diseases triggered by mitochondrial dysregulation-induced aberrant innate immune responses and potential therapeutic strategies.

Human disease	Target cells	Mechanism of action	Potential therapeutic strategies	Reference
Radiation-induced hematopoietic GI toxicity	Bone marrow cells, intestinal epithelial cells	AIM2 recognizes nucleus DNA damage triggered by radiation and caspase-1 dependent death	AIM2 inhibitor	Hu et al., 2016
Myocardial infarction	Macrophage	DAMPs from dying ischemic cells trigger cGAS-IRF3-interferon signaling axis	Inhibition of cGAS-IRF3 signaling pathway or inhibition of DAMPs release	King et al., 2017
Heart failure	Cardiomyocyte	mtDNA activates TLR9 mediated inflammatory responses	Blockage of TLR9 signaling	Oka et al., 2012
Atherosclerosis	Foam cells, macrophages	NLRP3 activation; NLRP3 facilitates mtDNA oxidative damage	Blockage of NLRP3 signaling pathway	Yu et al., 2013; Zheng et al., 2014; Shi et al., 2015; Afrasyab et al., 2016; Lin et al., 2018
	Macrophage	CD36 coordinates the intracellular conversion of endogenous ligands (such as oxLDL) into crystals or fibrils, which result in lysosomal disruption and NLRP3 inflammasome activation	CD36 inhibitor	Py et al., 2013; Sheedy et al., 2013
	Macrophage	8-oxoguanine glycosylase (OGG1) deficiency leads to oxidized mDNA, cyto c release, apoptosis, and IL-1 secretion	Removal of oxidative DNA	Tumurkhuu et al., 2016
STING-associated vasculopathy with onset in infancy (SAVI)	Endothelial cells	Gain of function mutation of STING leading to JAK activation	JAK inhibitor	Liu et al., 2014
Obesity and insulin resistance	Endothelial cells and adipose tissue	Obese caused mitochondrial damage and mtDNA leakage activate STING-IRF3 pathway	STING inhibitor	Mao et al., 2017

During early atherosclerosis, endothelial cells are activated by the elevation of ATP synthesis-uncoupled but proton leak-coupled mtROS without causing mitochondrial damage and EC death (Li et al., 2016, 2017), highlighting a novel regulatory network triggered by mtROS among mitochondrial metabolism, physiological EC activation, patrolling cell migration, and pathological inflammation. This suggests that mitochondrial antioxidants are promising therapies for vascular inflammation and cardiovascular diseases. Indeed, during the progression of atherosclerosis, excessive oxidative stress, dysfunctional mitochondria (Victor et al., 2009), ER stress (Chistiakov et al., 2014), and lysosome rupture (Yuan et al., 2000) are considered as one of the critical driver responsible for inflammasome activation. Clinical evidence has shown that the level of NLRP3 from peripheral blood monocyte is highly associated with the severity of coronary atherosclerosis in patients (Afrasyab et al., 2016), especially NLRP3 inflammasome has been observed to localize in the cytoplasm of foam cells and macrophages (Shi et al., 2015). Blockage of NLRP3 signaling inhibits the progression of atherosclerosis in apolipoprotein E-deficient (ApoE^−/−^) mice treated with a high-fat diet. NLRP3 knockdown also reduces macrophages and lipids while increases smooth muscle cells and collagen deposition of the plaque, contributing to plaque stabilization (Zheng et al., 2014). Interestingly, hydrogen sulfide which has shown to have anti-oxidative properties is able to also attenuate oxidative stress induced NLRP3 inflammasome activation via S-sulfhydrating c-Jun in macrophages (Lin et al., 2018). The priming event of NLRP3 is initiated by the cooperation of CD36 and a heterodimer of TLR4–TLR6 to convert intracellular ligands to crystals or fibrils followed by lysosomal disruption and NLRP3 activation (Sheedy et al., 2013). CD36 is an archetypal PRR which has been involved in the pathogenesis of atherosclerosis via modified endogenous danger signals such as oxidized low density lipoprotein (LDL) (oxLDL). CD36^−/−^ApoE^−/−^ mice had a reduced serological IL-1β and plaque cholesterol crystal accumulation. TLR and ROS signaling can increase the levels of NLRP3 via BRCA1/BRCA2-containing complex subunit 3 (BRCC3) which mediates deubiquitination of the LRR domain of NLRP3 and such modification is essential for its activation (Py et al., 2013). NLRP3 might also facilitate atherosclerosis via sensing oxidative mtDNA (Tumurkhuu et al., 2016). Increased mtDNA damage has been indicated in human atherosclerotic plaques compared to normal vessels. Further, leukocyte mtDNA damage was associated with higher-risk plaques in humans (Yu et al., 2013). 8-oxoguanine glycosylase (OGG1) is one of the major DNA glycosylase that is responsible for removing oxidative DNA. Ogg1 deficiency in atherosclerotic mice induces larger plaque and greater amount of lipid content, while both knockout of Ogg1 and NLRP3 rescue the enhanced atherosclerosis observed in Ogg1^−/−^ mice. Specifically, Ogg1^−/−^ macrophages showed increased oxidized mtDNA, cytochrome c, apoptosis, and IL-1 secretion. All these studies highlight NLRP3 as a potential therapeutic target for atherosclerosis (Tumurkhuu et al., 2016).

Chronic inflammation of endothelial cells initiates cardiovascular disease (Liao, 2013). Mitochondrial damage induced by palmitic acid and mitochondrial DNA release activates the STING–IRF3 pathway, which further trigger ICAM-1 expression and endothelial inflammation. Clinical evidence also identified STING-associated vasculopathy with onset in infancy (SAVI), an autoinflammatory disease result from gain-of-function mutations of STING (Liu et al., 2014). With the identified STING expression, endothelial cells exposed with cGAMP, the STING ligand, result in cellular activation indicated by elevated inducible nitric oxide synthase (iNOS) and E-selectin followed by apoptosis. Since mutant STING increased level of phosphorylated signal transducer and activator of transcription 1 (STAT1), the elucidation of such mechanism also suggests Janus kinase (JAK) inhibitor as a potential therapeutic strategy for SAVI, which is currently under clinical assessment. STING pathway is also involved in adipose tissue inflammation and insulin resistance. In obese mice triggered by high-fat diet, STING was found in adipose tissue and was actively involved in tissue inflammation and insulin resistance (Mao et al., 2017).

## Conclusion

Mitochondria are not only house machineries that support cellular essential activities, but also important sources of endogenous DAMPs including ROS as well as necessary triggers for inflammasome signaling. A large number of evidence has emerged linking dysfunctional mitochondria to aberrant innate immune responses. Nevertheless, our understanding of precise roles the inflammasomes in response to mitochondrial malfunction and ROS are still lacking. Many significant questions regarding the molecular machineries which initiate inflammasome activation upon mitochondria disorder and ROS remain to be addressed. Further elucidation of the interplay of ROS, mitochondrial function and inflammasome pathways might open up a new horizon for the development of immunotherapeutic strategy for chronic inflammation diseases such as cardiovascular diseases.

## Author Contributions

YC, ZZ, and WM wrote the paper.

## Conflict of Interest Statement

The authors declare that the research was conducted in the absence of any commercial or financial relationships that could be construed as a potential conflict of interest.
